# Supplementary material for: An autologous cell-based therapeutic vaccine expressing IL6/1 fusokine drives robust anti-tumor response against ovarian cancer
Source: bioRxiv. 2026 May 8:2026.05.05.721149. Preprint. [Version 1] doi: 10.64898/2026.05.05.721149 (PMC13174554; doi:10.64898/2026.05.05.721149)
Supplement: Supplement 1 [file media-1.pdf]

Supplementary Figure 1:

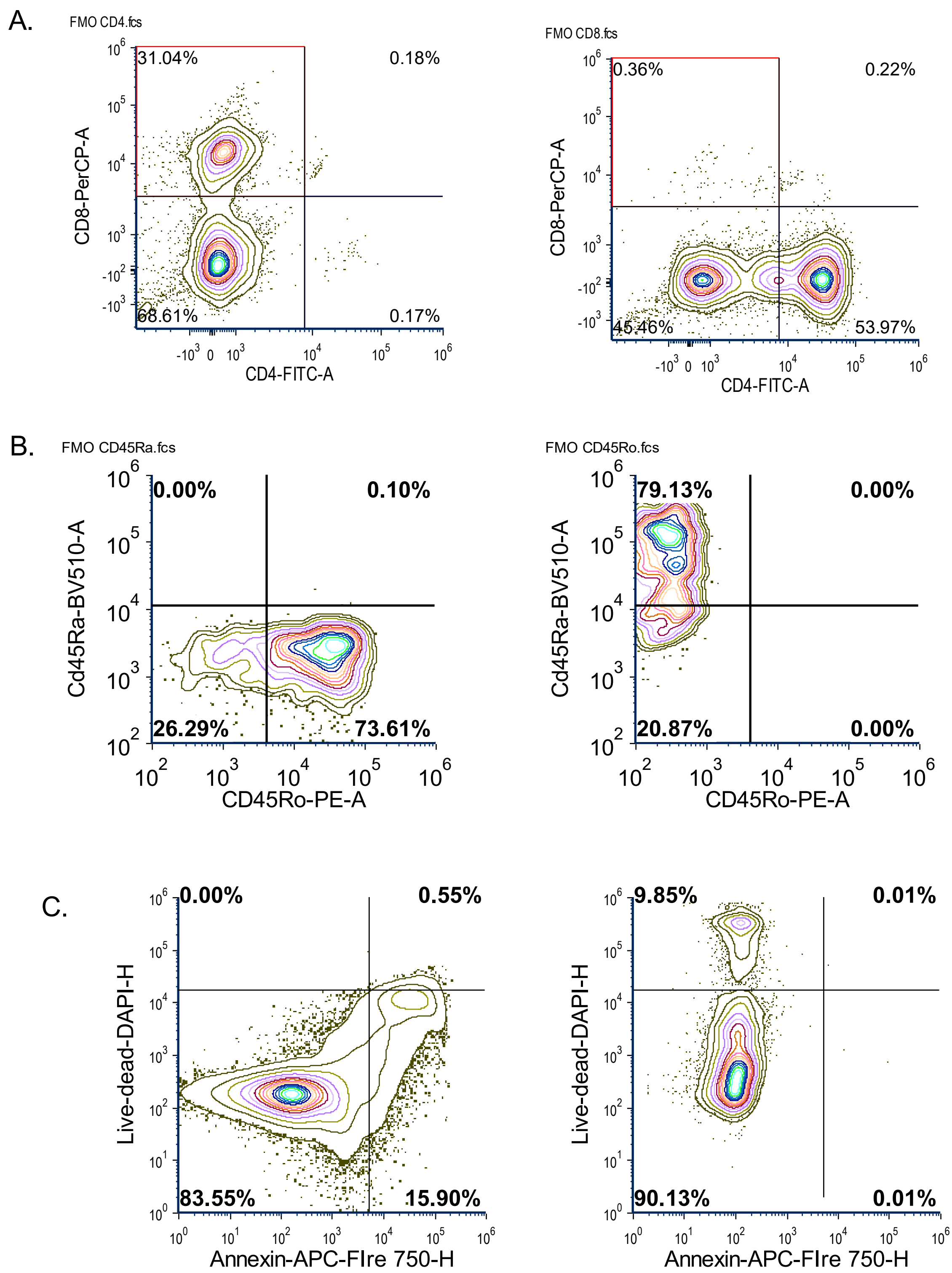

Supplementary Figure 1: (A) FMOs for CD4 and CD8 T cell proliferation data. (B) FMOs for CD45Ra and CD45Ro T cell data. (C) FMOs for DAPI and Annexin v for T cell apoptosis data.

Supplementary Figure 2:

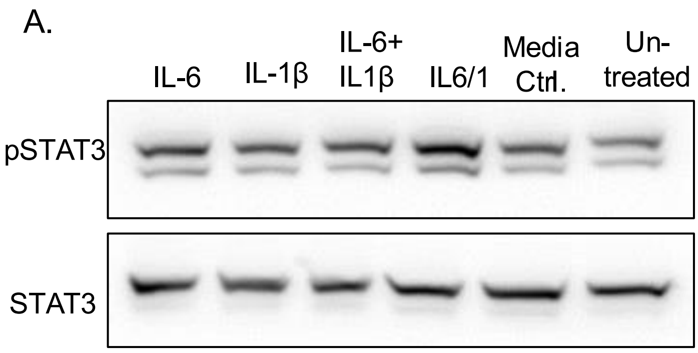

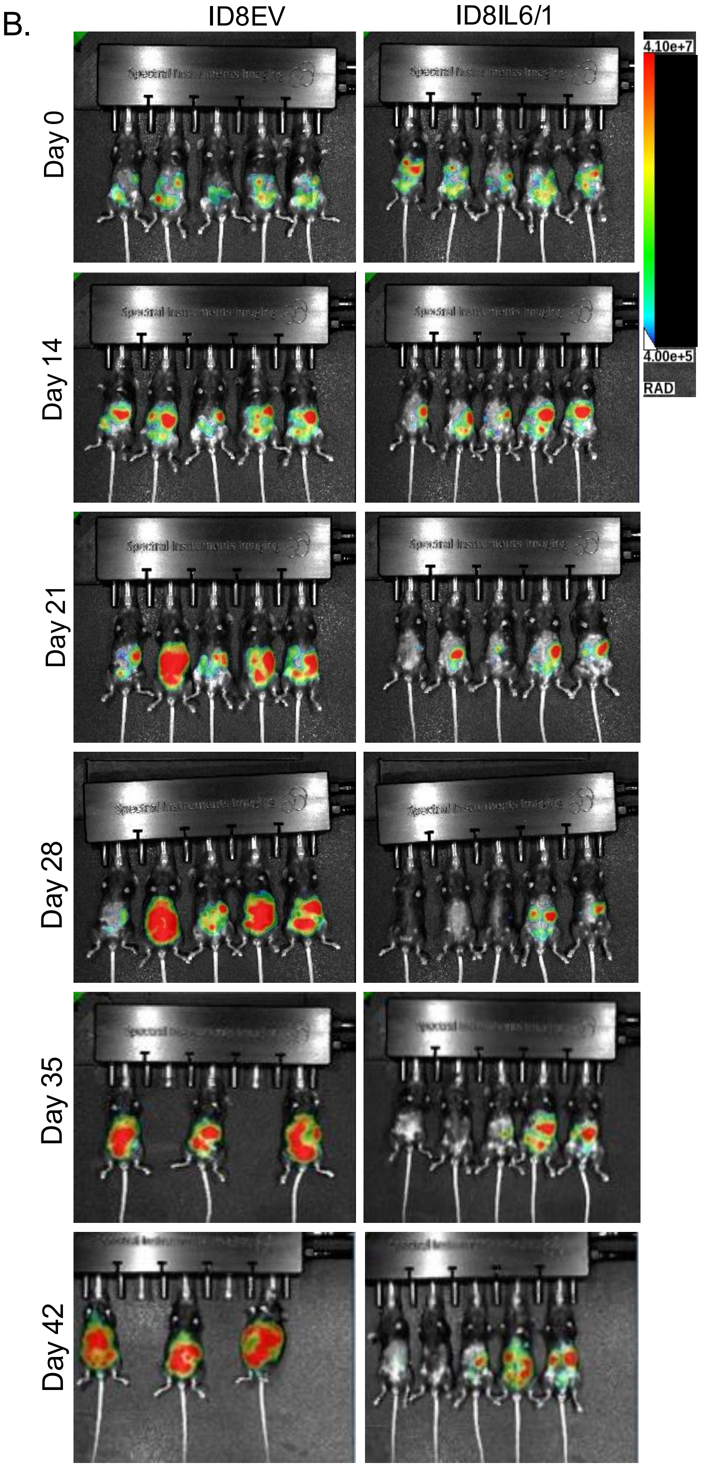

Supplementary Figure 2: (A) Human PBMCs derived CD3 T cells were induced to proliferate in the presence of equivalent amount of IL-6. IL1β, IL-6 + IL1β, IL6/1. After 72 h of culturing, cells were collected, lysed and subjected to western blot. (B) Tumor growth was quantitatively analyzed using bioluminescence (BLI) signals obtained from LAGO spectrum at a week after tumor inoculation and subsequent weeks. Longitudinal images of a representative mouse from each treatment group were presented from a week after tumor inoculation before treatment and subsequent weeks.
